# Evaluation of the *Enterococcus faecalis* Biofilm-Associated Virulence Factors AhrC and Eep in Rat Foreign Body Osteomyelitis and *In Vitro* Biofilm-Associated Antimicrobial Resistance

**DOI:** 10.1371/journal.pone.0130187

**Published:** 2015-06-15

**Authors:** Kristi L. Frank, Paschalis Vergidis, Cassandra L. Brinkman, Kerryl E. Greenwood Quaintance, Aaron M. T. Barnes, Jayawant N. Mandrekar, Patrick M. Schlievert, Gary M. Dunny, Robin Patel

**Affiliations:** 1 Department of Microbiology, University of Minnesota, Minneapolis, Minnesota, United States of America; 2 Division of Clinical Microbiology, Department of Laboratory Medicine and Pathology, Mayo Clinic, Rochester, Minnesota, United States of America; 3 Division of Biomedical Statistics and Informatics, Mayo Clinic, Rochester, Minnesota, United States of America; 4 Division of Infectious Diseases, Department of Internal Medicine, Mayo Clinic, Rochester, Minnesota, United States of America; University Medical Center Utrecht, NETHERLANDS

## Abstract

*Enterococcus faecalis* can cause healthcare-associated biofilm infections, including those of orthopedic devices. Treatment of enterococcal prosthetic joint infection is difficult, in part, due to biofilm-associated antimicrobial resistance. We previously showed that the *E*. *faecalis* OG1RF genes *ahrC* and *eep* are *in vitro* biofilm determinants and virulence factors in animal models of endocarditis and catheter-associated urinary tract infection. In this study, we evaluated the role of these genes in a rat acute foreign body osteomyelitis model and in *in vitro* biofilm-associated antimicrobial resistance. Osteomyelitis was established for one week following the implantation of stainless steel orthopedic wires inoculated with *E*. *faecalis* strains OG1RF, Ω*ahrC*, and ∆*eep* into the proximal tibiae of rats. The median bacterial loads recovered from bones and wires did not differ significantly between the strains at multiple inoculum concentrations. We hypothesize that factors present at the infection site that affect biofilm formation, such as the presence or absence of shear force, may account for the differences in attenuation in the various animal models we have used to study the Ω*ahrC* and ∆*eep* strains. No differences among the three strains were observed in the planktonic and biofilm antimicrobial susceptibilities to ampicillin, vancomycin, daptomycin, linezolid, and tetracycline. These findings suggest that neither *ahrC* nor *eep* directly contribute to *E*. *faecalis* biofilm-associated antimicrobial resistance. Notably, the experimental evidence that the biofilm attachment mutant Ω*ahrC* displays biofilm-associated antimicrobial resistance suggests that surface colonization alone is sufficient for *E*. *faecalis* cells to acquire the biofilm antimicrobial resistance phenotype.

## Introduction

Infection is an infrequent but serious complication of orthopedic implants [1]. Bacteria involved in prosthetic joint infection (PJI) grow as biofilms on prosthesis surfaces and in surrounding tissues [2]. Biofilm growth confers enhanced resistance to antimicrobial agents and the host immune response, making biofilm-associated infections like PJI difficult to treat clinically [3].

Approximately 65% of prosthetic hip and knee infections are caused by Gram-positive cocci, with *Staphylococcus aureus* and coagulase negative staphylococci comprising the majority of these infections [1, 4]. Another genus of Gram-positive cocci, *Enterococcus*, is associated with approximately 2.5% of PJI cases [5, 6]. *Enterococcus faecalis* is the most frequently identified species in enterococcal PJI [6, 7]. Substantial details on the genetic and regulatory mechanisms that contribute to staphylococcal biofilm formation on implant surfaces have been elucidated and were reviewed recently [8]. In contrast, there are no published data available on genetic mechanisms of *E*. *faecalis* biofilm formation involved in orthopedic implant infection. Increasing our knowledge in this area is clinically important because prolonged antimicrobial therapy and surgical interventions to remove infected implanted devices are frequently required for clinical treatment of enterococcal PJI, and treatment failure rates as high as 44% have been reported [5–7, 9]. Furthermore, understanding genetic determinants of biofilm formation outside of staphylococci may provide insights that will generalize to other organisms.

Significant efforts to understand the genetic basis of *E*. *faecalis* biofilm formation have been undertaken over the past decade [10]. The *E*. *faecalis* collagen-binding protein Ace, the endocarditis- and biofilm-associated pilus (Ebp), and the secreted protease gelatinase (GelE) are well-studied biofilm determinants encoded on the chromosome that have characterized roles in animal models of biofilm-associated infection [11–15]. In order to identify additional biofilm determinants in the core genome of *E*. *faecalis*, our group performed two independent genetic screens with strain OG1RF [16, 17], a well-studied strain lacking plasmids or other mobile genetic elements [18]. From among the 78 distinct genetic loci implicated as biofilm determinants in these screens [16, 17], we showed in two recent experimental animal infection studies that the genes *ahrC* and *eep* are biofilm-associated virulence factors [19, 20]. The *ahrC* gene encodes an ArgR family transcriptional regulator critical for *E*. *faecalis* attachment to both *in vitro* polystyrene and *ex vivo* porcine heart valve surfaces during the early stages of biofilm formation [17, 19, 21]. The *eep* gene encodes a membrane metalloprotease that is a member of the site 2 protease family, a conserved class of enzymes that perform regulated intramembrane proteolysis as part of the processing of signals detected at the cell membrane [22]. Eep was first described for its role in generating peptide pheromones that act as signaling molecules in conjugative plasmid transfer [23]. More recently, we demonstrated Eep to be instrumental in *E*. *faecalis* biofilm maturation, as deletion of *eep* in OG1RF causes alterations in microcolony architecture and extracellular matrix distribution [19, 20]. As virulence factors, both *ahrC* and *eep* are major contributors to infection in a rabbit model of experimental endocarditis, while *ahrC* plays a more significant role than *eep* in a murine catheter-associated urinary tract infection (UTI) model [19, 20]. A major difference between these infection models is that the catheter-associated UTI model involves a foreign body. We hypothesize that the function of *ahrC* in biofilm attachment is particularly important in the pathogenesis of *E*. *faecalis* foreign body infection.

Although collections of *E*. *faecalis* isolates from orthopedic device infections have been analyzed for virulence characteristics such as *in vitro* biofilm formation ability and gelatinase production [24, 25], there have been no studies to date examining the roles of specific *E*. *faecalis* genetic determinants in orthopedic device-associated infection. The relationship between *E*. *faecalis* biofilm-associated genes and biofilm-induced antimicrobial resistance is also undefined. In the present study, we established a rat model of *E*. *faecalis* acute foreign body osteomyelitis to evaluate how *ahrC* and *eep* affect biofilm formation in the context of bone infection. We then compared the antimicrobial susceptibilities of planktonic and biofilm forms of OG1RF with those of strains with disruptions in either *ahrC* or *eep* to determine if these genetic determinants contribute to *E*. *faecalis* biofilm-specific antimicrobial resistance.

## Methods

### Bacterial strains

*E*. *faecalis* OG1RF [26] and two derivative strains harboring gene disruptions were studied. OG1RF Ω*ahrC* (previously called mutant 20K19 [17]) contains a transposon insertion in *ahrC* at locus OG1RF_10717 (EF0983). OG1RF Δ*eep* (previously called JRC106 [27]) has an in-frame deletion in *eep* at locus OG1RF_11819 (EF2380).

### Animal experiments

#### Ethics statement

Animal experiments were carried out according to protocols approved by the Mayo Clinic Institutional Animal Care and Use Committee (approval number A37013). Animals did not experience difficulty with locomotion and did not demonstrate signs of systemic infection. Surgical sites were checked daily to ensure healing. Animals received acetaminophen (1.28 mg/ml) in drinking water 48 hours prior to surgery and for five days following surgery. Buprenorphine slow release (0.6 mg/kg) was administered subcutaneously immediately prior to the start of surgery or immediately following completion of surgery while animals were still anesthetized.

#### Inoculum preparation

Bacterial strains were grown in trypticase soy broth (TSB) at 37°C with 5% CO_2_. Cultures at a 1.0 McFarland density were prepared and used to inoculate wires. Alternatively, cultures at 1.0 McFarland density were serially diluted to the specified cell densities for experiments using lower inocula. 1 mm diameter stainless steel grooved Kirschner wires (Zimmer, Warsaw, IN), measuring 5 mm in length, were submerged in the prepared inocula and incubated at 37°C for two hours.

To confirm that wire inocula were in the target range, matched wire samples were removed from the inoculum, placed in fresh TSB, vortexed, sonicated, and quantitatively cultured as previously described [28]. Four wires were assayed per experiment and the experiment was performed twice. Measurements from all wires were averaged together.

#### Rat model of acute foreign body osteomyelitis

Foreign body osteomyelitis was established in male Wistar rats. We modified our previously developed chronic model [28] as follows: wires were inoculated prior to implantation into the proximal tibia and bone was harvested after one week. The left legs of rats were shaved with an electric clipper, and the skin disinfected with povidone-iodine or Hibiclens (Mölnlycke Health Care US, LLC, Norcross, GA). Surgical anesthesia was induced with 60 mg/kg ketamine (Ketaset, Fort Dodge, IA) and 6 mg/kg xylazine (Vettek, Phoenix Scientific Inc., St. Joseph, MO). The proximal third of the left rat tibia was surgically exposed, and a 3 mm hole drilled into the medullary cavity. A wire inoculated with one of the strains was implanted into the exposed tibial cavity, and the hole was covered with dental gypsum. The muscle was closed with 3–0 vicryl (Ethicon, Inc., Sommerville, NJ) using simple continuous sutures, and then the skin was closed with tissue glue and surgical clips and sprayed with antiseptic.

One week after implantation, rats were sacrificed using CO_2_ and the left tibiae were aseptically removed. Tibiae were frozen to -70°C and cryopulverized. Cryopulverized bone was suspended in 2 ml TSB, vortexed, sonicated, serially diluted, and plated onto sheep blood agar plates for quantitative cultures. TSB-suspended cryopulverized bones were stored at -70°C. If there was no growth in quantitative cultures, aliquots of TSB-suspended bone were incubated for 48 hours to assess bacterial recovery qualitatively. Wires extracted from cryopulverized bone were placed separately in 1 ml TSB, vortexed, sonicated, and cultured for both quanitative and qualitative assessment of bacterial growth [28].

#### *In vivo* re-inoculation of Ω*ahrC* strains passaged once through the rat osteomyelitis model

Two *E*. *faecalis* isolates recovered from rat osteomyelitis were obtained by streaking aliquots of TSB-suspended cryopulverized bones stored at -70°C for isolation on sheep blood agar plates. A single colony of either isolate was used to inoculate cultures that subsequently served as the infection inoculum. Inoculum preparation and rat osteomyelitis infections were performed as described above.

### Microtiter plate biofilm assay

*E*. *faecalis* isolates recovered from rat osteomyelitis were obtained by streaking aliquots of TSB-suspended bones for isolation on brain heart infusion agar. Microtiter plate biofilm assays were carried out as described previously [19]. Four to eight biological replicates of *E*. *faecalis* isolates recovered from the rat osteomyelitis model were tested.

### Antimicrobial susceptibility testing

The minimum inhibitory concentrations (MIC) of ampicillin, vancomycin, daptomycin, linezolid, and tetracycline were determined by broth microdilution [29]. *E*. *faecalis* ATCC 29212 served as a control strain. The minimum bactericidal concentration (MBC) was defined as the highest dilution showing ≥ 99.9% killing [30].

Biofilm antimicrobial susceptibilities were tested as previously described [31]. Briefly, biofilms were grown on the surface of pegs immersed in the wells of a microtiter plate in TSB. After incubation, the pegged lid was rinsed three times in normal saline to remove planktonic bacteria and transferred to a second plate containing serial dilutions of each antimicrobial agent in cation-adjusted Mueller-Hinton broth (CAMHB). The plate was incubated at 37°C for another 20 hours (inhibitory plate). The minimum biofilm inhibitory concentration (BIC) was defined as the minimum concentration required to prevent growth in the inhibitory plate. The pegged lid was again rinsed three times in normal saline and transferred to a 96-well plate filled with CAMHB. The plate was sonicated (40 kHz, 320 mW/cm^2^) for 5 minutes in a bath sonicator (Zenith, Norwood, NJ) to dislodge biofilms from pegs. The optical density of the wells was measured on a plate reader at 600 nm (OD_600_) before and after incubation for 24 hours at 37°C (recovery plate). The minimum biofilm bactericidal concentration (BBC) was defined as the lowest concentration of drug resulting in an OD_600_ difference at or below 10% of the positive control well reading.

### Statistical methods

Quantitative culture results were expressed as log_10_ CFU per gram of bone or cm^2^ of wire. For statistical purposes, we considered absence of growth as 0.1 log_10_ CFU per gram of bone or cm^2^ of wire and qualitative growth from broth only as 0.5 log_10_ CFU per gram of bone or cm^2^ of wire. The Wilcoxon rank sum test was used to compare the results of quantitative cultures in bone and wire between OG1RF and each of the mutants. Biofilm formation by parent and rat-passaged OG1RF or ∆*eep* isolates was compared with unpaired t-tests. The Kruskal-Wallis test with Dunn’s multiple comparison post-hoc test was used in experiments with varying concentrations of inocula. All tests were two-sided and *p*-values < 0.05 were considered statistically significant.

## Results

### Evaluation of *E*. *faecalis* OG1RF, Ω*ahrC*, and Δ*eep* in a rat model of acute foreign body osteomyelitis

**Fig 1:**
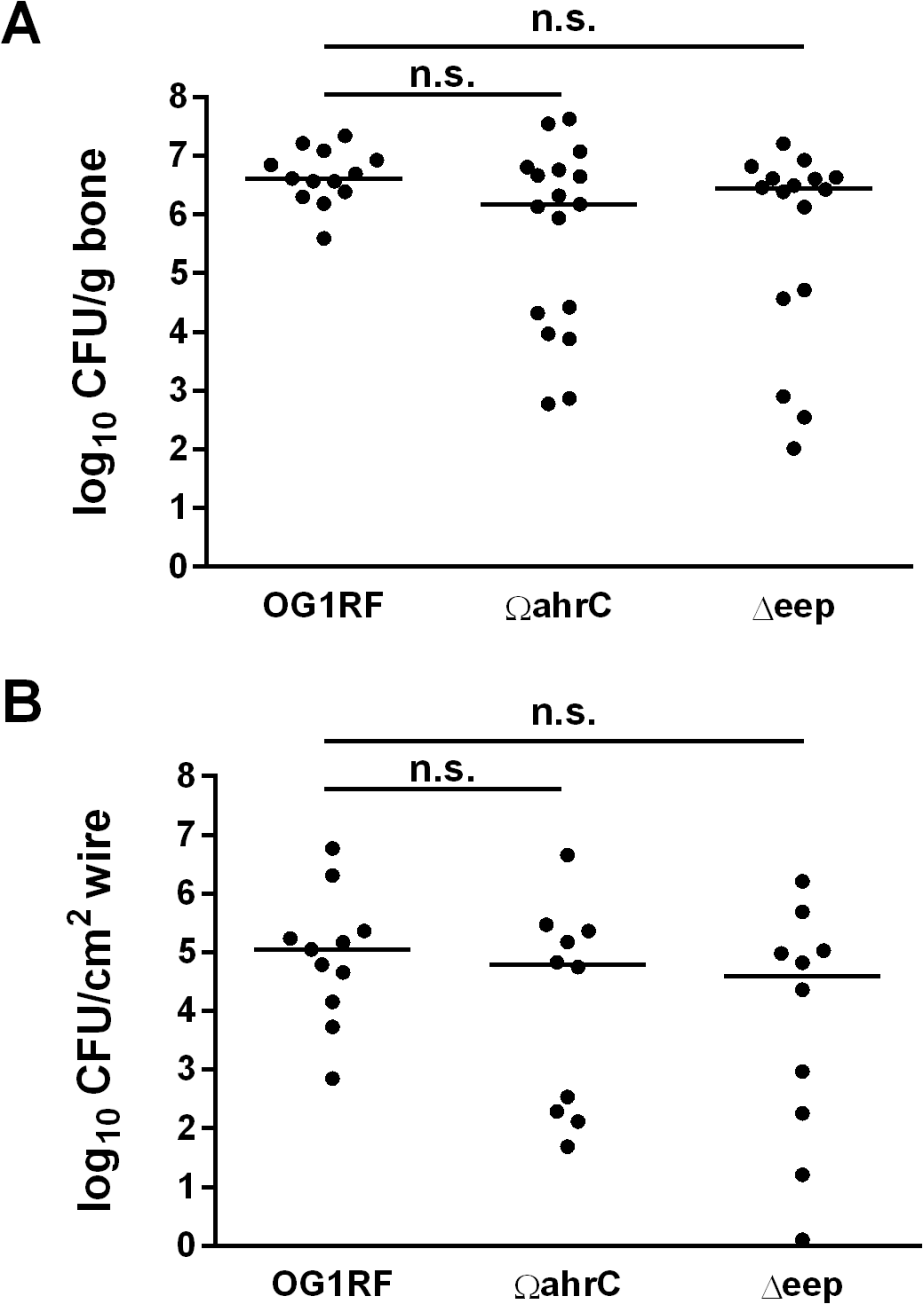
Quantitative bone (A) and wire (B) culture results from rats infected with *E*. *faecalis* OG1RF and gene disruption mutants Ω*ahrC* and Δ*eep*. Wires were submerged in bacterial cultures at a density of 1.0 McFarland for two hours prior to surgical implantation as described in the Methods section. Infections were carried out for one week. Bacterial loads are given as log_10_ CFU per gram of bone or cm^2^ of wire surface. Solid horizontal lines represent median values. The Wilcoxon rank sum test was used to compare the results of quantitative cultures in bone and wire between OG1RF and each of the mutants in (A) and (B). n.s., not significant. (A) OG1RF, n = 13; Ω*ahrC*, n = 17; Δ*eep*, n = 16. (B) OG1RF, n = 11; Ω*ahrC*, n = 10; Δ*eep*, n = 10. Wire counts were not performed on all animals shown in (A) due to their use in other (unrelated) studies.

We evaluated the contributions of *ahrC* and *eep* to the infection process in an experimental rat foreign body osteomyelitis model. Wires were submerged in cultures of OG1RF, Ω*ahrC*, or Δ*eep* for two hours prior to implantation in rat tibiae. The resultant average (standard deviation) log_10_ CFU/cm^2^ wire colonization loads were 6.43 (0.24), 6.35 (0.36), and 6.36 (0.33) for OG1RF, Ω*ahrC*, and ∆*eep*, respectively, confirming that similar bacterial loads of all strains were introduced at the site of infection. Following wire implantation, infections were carried out for one week to assess the abilities of the three strains to colonize both bone and implanted wires. The results of quantitative cultures for bones and wires harvested from the infected animals are shown in Fig 1. Median bacterial quantities from bones or wires were not significantly different between animals infected with OG1RF or either mutant, indicating that neither disruption of *ahrC* nor deletion of *eep* was sufficient to reduce the bacterial burden in this model following one week of infection.

### The bimodal distribution of bone bacterial loads in rats infected with biofilm mutants is not the result of *in vivo* selection of isolates with enhanced abilities to form biofilms

**Fig 2:**
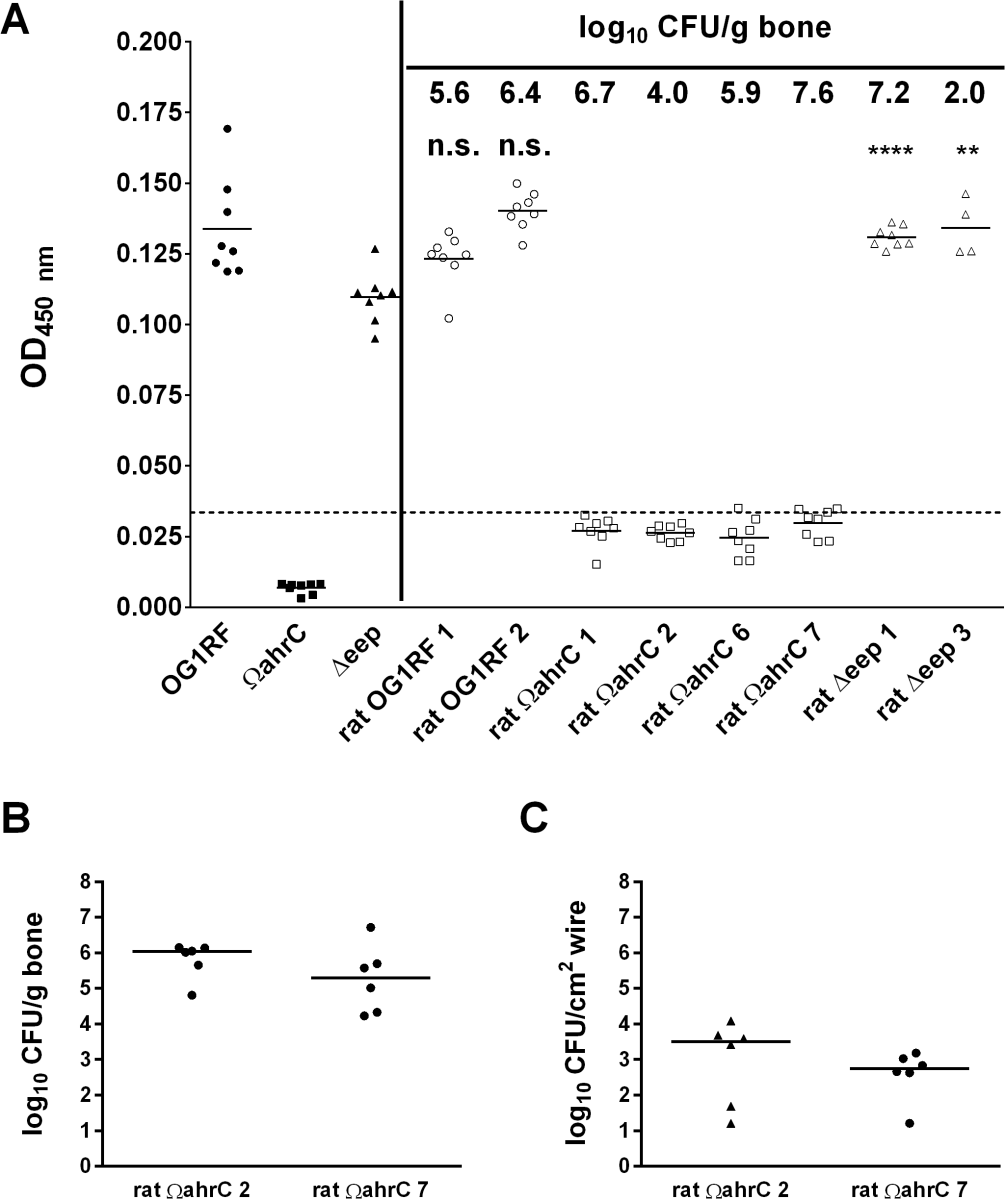
Passage through the rat foreign body osteomyelitis model does not affect *in vitro* biofilm formation (A) and degree of bone or wire colonization upon re-inoculation into the infection model (B). (A) Select *E*. *faecalis* isolates recovered from rat foreign body osteomyelitis experiments shown in Fig 1 were assayed for *in vitro* biofilm formation ability. The black symbols to the left of the vertical line show biofilm formation by independent cultures started with individual colonies of the parent OG1RF, Ω*ahrC*, and Δ*eep* isolates that had not been passaged through the rat foreign body osteomyelitis model. The open symbols to the right of the vertical line show biofilm formation by independent cultures inoculated from individual colonies of isolates of the three strains recovered from rat foreign osteomyelitis bone samples. The bacterial load of each bone sample (log_10_ CFU/g bone) from which the tested isolates were recovered is indicated at the top. Solid horizontal lines indicate means. The dashed horizontal line indicates 75% below the average biofilm biomass for the OG1RF parent strain. Isolates with means below the dashed line were deemed to be deficient in biofilm formation. Unpaired t-tests were used to compare biofilm formation by rat-passaged OG1RF or ∆*eep* isolates (right of vertical line) with the respective parent isolates (left of vertical line). n.s., not significant; ****, *p*-value < 0.0001; **, *p*-value < 0.01. (B) Quantitative bone culture results given as log_10_ CFU per gram of bone from rats infected with *E*. *faecalis* Ω*ahrC* isolates recovered from rat infections shown in Fig 1. The isolate designated as rat Ω*ahrC* 2 was recovered from an animal with a bone bacterial load of 4.0 log_10_ CFU/g bone. The isolate designated as rat Ω*ahrC* 7 was recovered from an animal with a bone bacterial load of 7.6 log_10_ CFU/g bone. (C) Quantitative wire culture results given as log_10_ CFU per cm^2^ of wire surface from rats infected with *E*. *faecalis* isolates rat Ω*ahrC* 2 and rat Ω*ahrC* 7, which were recovered from the animal infections shown in Fig 1. In (B) and (C), horizontal lines represent the medians.

Although there were no differences in the median quantities among the three strains, we were interested in the observation that the bone bacterial loads from both mutant strains exhibited a bimodal distribution, while the results from OG1RF exhibited a unimodal distribution (Fig 1A). Specifically, the bacterial loads of 6/17 bones (35%) infected with Ω*ahrC* and 5/16 bones (31%) infected with Δ*eep* occurred in lower populations. The bimodal distributions suggested that the mutant strains might have been subjected to selective pressures *in vivo* that resulted in a population of cells with increased capacity to cause infection in the acute foreign body osteomyelitis model. Since the Ω*ahrC* and Δ*eep* strains have documented phenotypic aberrancies in biofilms grown under *in vitro* conditions [17, 19, 20], and biofilm formation is key to the pathogenesis of prosthetic joint infection [2], we tested selected bone isolates from the initial rat infections (Fig 1A) for biofilm formation in a microtiter plate biofilm assay (Fig 2A). We specifically tested isolates of Ω*ahrC* and Δ*eep* from both high- and low-colonized bone samples (Fig 1A). Average biofilm formation for OG1RF after passage through the rat was unchanged relative to the parent isolate. Although there was a measureable increase in the OD_450_ for all rat-passaged isolates of Ω*ahrC* compared to the Ω*ahrC* parent isolate, the average biofilm formation for all isolates fell more than 75% below the average biofilm biomass for the OG1RF parent isolate (dashed line in Fig 2A). Therefore, all isolates of Ω*ahrC* tested in this assay were deemed to be deficient in biofilm formation, which is consistent with our previous work [17, 19]. Both rat-passaged Δ*eep* isolates produced statistically significant higher amounts of stainable biomass, indicating this property was independent of whether the isolates originated from high- or low-colonized bone samples. Overall, these data suggest that the bimodal data distributions for Ω*ahrC* and Δ*eep* shown in Fig 1 are not the result of *in vivo* selection of isolates with enhanced biofilm formation abilities.

To further assess whether the bimodal distributions may have been caused by inherent variances in the infection model, isolates of Ω*ahrC* from bones with bacterial loads of 4.0 and 7.6 log_10_ CFU/g bone were re-inoculated into rats. All of the harvested bone samples had similar bacterial levels (Fig 2B), while the bacterial quantities recovered from wires were more variable (Fig 2C). Since the isolates used for re-inoculation originated from the different subpopulations of the bimodal population and produced similar results, the bimodal effect observed in Fig 1 is more likely due to inherent properties of the model itself rather than selection of genetically distinct subpopulations.

### Evaluation of the inoculum size needed to establish infection

**Fig 3:**
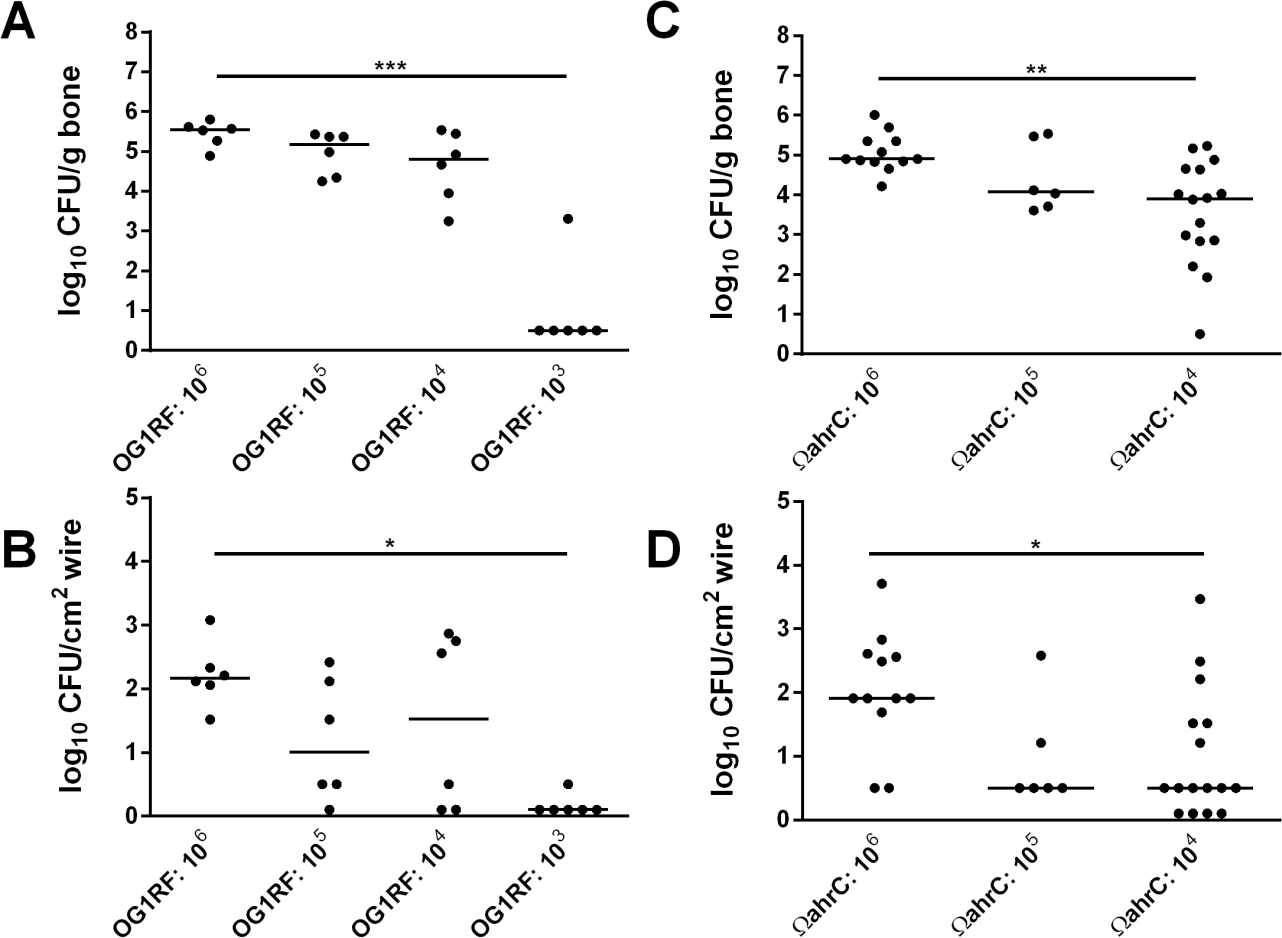
Quantitative bone (A, C) and wire (B, D) culture results from rats infected with decreasing concentrations of *E*. *faecalis* OG1RF (A, B) or the transposon disruption mutant Ω*ahrC* (C, D). The approximate CFU/cm^2^ wire inoculum used for each set of infected rats is given on the x-axis as a power of 10. Bacterial quantities are given as the log_10_ CFU per gram of bone or cm^2^ of wire surface recovered from rats one week post-infection (y-axis). Horizontal lines represent median values. The Kruskal-Wallis test with Dunn’s multiple comparison post-hoc test was used to determine significance across inocula groups in each panel. ***, *p*-value < 0.001; **, *p*-value < 0.01; *, *p*-value < 0.05.

We next examined whether the biofilm mutant strain Ω*ahrC* differed from OG1RF in the inoculum size required to consistently colonize damaged bones and implanted wires. To test this, we implanted wires inoculated with decreasing concentrations of OG1RF and Ω*ahrC* in rats and carried out infection experiments as described above. The bone and wire bacterial loads recovered from rats inoculated with OG1RF concentrations at 10^3^ CFU/cm^2^ wire were significantly decreased compared to the 10^6^ CFU/cm^2^ wire inoculum (Fig 3A and 3B). In contrast, bone and wire colonization by the Ω*ahrC* strain was significantly lower with 10^4^ CFU/cm^2^ wire compared to the 10^6^ CFU/cm^2^ wire inoculum (Fig 3C and 3D). Despite this, the difference in bone bacterial loads between OG1RF and the Ω*ahrC* strain at the 10^4^ CFU/cm^2^ inoculum trended toward, but did not reach, statistical significance (*p*-value = 0.059 by Wilcoxon rank sum test; compare OG1RF: 10^4^ in Fig 3A with Ω*ahrC*: 10^4^ in Fig 3C). There was no difference in bacterial cell recovery from the wires for the same two strains at the 10^4^ inoculum (*p*-value = 0.562 by Wilcoxon rank sum test; compare OG1RF: 10^4^ in Fig 3B with Ω*ahrC*: 10^4^ in Fig 3D).

### Planktonic and biofilm antimicrobial susceptibilities

Finally, we investigated whether Ω*ahrC* and Δ*eep* play a role in *E*. *faecalis* biofilm-associated antimicrobial resistance. Minimum inhibitory and bactericidal activities of ampicillin, vancomycin, daptomycin, linezolid, and tetracycline against planktonic and biofilm forms of OG1RF, Ω*ahrC*, and Δ*eep* are shown in Table 1. OG1RF was susceptible to all antimicrobials tested on planktonic cells. No significant differences were observed between OG1RF and the mutant strains. Ampicillin and daptomycin exhibited bactericidal activity against planktonic cells *in vitro*. Vancomycin, linezolid, and tetracycline were bacteriostatic. In contrast, BBCs were uniformly higher than 128 μg/mL for all strains. Therefore, biofilms of OG1RF, Ω*ahrC*, and Δ*eep* are highly resistant to the antimicrobial agents tested.

**Table 1:** Antimicrobial minimum inhibitory and bactericidal concentration for *E*. *faecalis* OG1RF, Ω*ahrC*, and Δ*eep* grown in planktonic and biofilm forms.

	Antimicrobial agent, concentration (μg/mL)				
Strain	Ampicillin	Vancomycin	Daptomycin	Linezolid	Tetracycline
OG1RF					
MIC	0.5	2	0.5	2	2
MBC	1	>128	4	>128	>32
BIC	0.5	2	1	4	0.5
BBC	>128	>128	>128	>128	>128
Ω*ahrC*					
MIC	0.5	2	0.5	2	2
MBC	2	>128	2	>128	>32
BIC	0.5	4	1	4	1
BBC	>128	>128	>128	>128	>128
Δ*eep*					
MIC	0.5	2	2	4	2
MBC	0.5	>128	8	>128	>32
BIC	0.5	4	2	2	1
BBC	>128	>128	>128	>128	>128

Abbreviations: MIC, planktonic minimum inhibitory concentration; MBC, planktonic minimum bactericidal concentration; BIC, biofilm minimum inhibitory concentration; BBC, biofilm minimum bactericidal concentration.

## Discussion

The importance of biofilm formation in the pathogenesis of enterococcal infections is now widely recognized [24, 32–34]. We initially identified the *E*. *faecalis* genes *ahrC* and *eep* as biofilm formation genes in genetic screens that employed transposon mutagenesis and recombinase-based *in vivo* expression technology, respectively [16, 17]. AhrC is a transcription factor that affects surface attachment during early biofilm formation, while Eep is an intramembrane metalloprotease that contributes to the spatial distribution of microcolonies and extracellular matrix later in biofilm development [19]. Strains with disruptions in either gene demonstrated significant attenuation in a rabbit model of endocarditis, with each having a 4.0-log_10_ CFU reduction in valve colonization relative to the wild-type strain [19, 20]. The same strains yielded different results in a mouse model of catheter-associated UTI [19]: deletion of *ahrC* was detrimental for bacterial colonization of catheter implants, bladders, and kidneys, while deletion of *eep* only impaired kidney colonization. The results of these previously conducted animal infections indicate that both genes are virulence factors in *E*. *faecalis* biofilm-associated infections, yet suggest that *ahrC* may be more important than *eep* in the pathogenesis of infections involving a foreign body.

Here, we tested the ability of the enterococcal mutants to establish infection in a rat model of acute foreign body osteomyelitis (Fig 1). We implanted wires into rat bones after two hours of submersion in bacterial cultures. Surprisingly, the median bacterial loads recovered from bones and wires did not differ significantly between the strains (Fig 1). Further, the data from our subsequent *in vitro* biofilm and *in vivo* re-inoculation experiments (Fig 2) indicated that the bimodal populations that arose with the biofilm mutant strains in Fig 1 are probably not the result of *in vivo* selection of isolates with enhanced biofilm formation *in vitro* or *in vivo*. Foreign body osteomyelitis infections performed with decreasing inocula revealed that there was a statistically significant drop in bone colonization by the Ω*ahrC* strain between inocula at 10^6^ and 10^4^ CFU/cm^2^ (Fig 3B). This was 10-fold higher than OG1RF, where a statistically significant drop in bone colonization was not observed until 10^3^ CFU/cm^2^ (Fig 3A). However, the pair-wise comparison of OG1RF and Ω*ahrC* bone colonization at the 10^4^ CFU/cm^2^ inoculum did not reach statistical significance with the number of animals we used. We also carried out preliminary experiments to test decreasing concentrations of the ∆*eep* strain in the same experimental conditions used for OG1RF and Ω*ahrC* in Fig 3. The data obtained from the limited number of rats infected with ∆*eep*, which forms mature biofilms that are disorganized [19], were more similar to results obtained with OG1RF than Ω*ahrC* (data not shown).

Given that neither of the biofilm mutants with attenuation phenotypes in two other biofilm-associated infection animal models were attenuated in the rat foreign body osteomyelitis model used in this study, we hypothesize that factors present at the infection site that affect biofilm formation may contribute to the different phenotypes observed in the various animal models. In the previous animal models, endocarditis was established on mechanically-damaged heart valve tissue, and catheter-associated UTI involved the presence of a foreign body in an otherwise undisrupted urinary tract. Biofilm bacteria in either of these models were subjected to shear forces (e.g., blood or urine flow). In the osteomyelitis model, which involves both tissue damage and a foreign body, infection is established in damaged tissue (i.e., drilled bone) in the presence of a stainless steel wire. However, little to no shear force is present in the medullary cavity of the tibia. In addition, the foreign body osteomyelitis model represents a more localized infection relative to the endocarditis and catheter-associated UTI models, wherein the biofilm bacteria may be more likely to spread through the blood stream or ascend from the bladders to the kidneys. These differences may account for the lack of attenuation of the biofilm mutant strains in the rat foreign body osteomyelitis model used in this study and underscore the importance of taking the pathogenesis of biofilm infections into consideration when comparing strains between models.

We did not observe a difference in planktonic antimicrobial susceptibilities between the three strains (Table 1), indicating that the gene products of *ahrC* and *eep* do not affect the activities of the antimicrobial agents tested under the planktonic conditions evaluated. We also measured the BICs and BBCs of the three strains. The BIC represents the lowest concentration where bacteria shed from the biofilm cannot establish growth. BBCs determine the minimum antimicrobial concentration where biofilm bacteria are not viable; thus, BBCs represent killing concentrations. The highest antimicrobial concentrations tested were insufficient to eradicate biofilms of any of the strains (Table 1). These findings provide evidence for the concept that complex mechanisms are involved in biofilm-specific antimicrobial resistance, where the absence of a single genetic determinant does not result in increased antimicrobial susceptibility [35]. The observation that the Ω*ahrC* strain, which fails to progress past the attachment phase of biofilm maturation [17, 19], displays biofilm antibiotic resistance is notable. This suggests that surface attachment alone is sufficient for *E*. *faecalis* cells to acquire the biofilm antimicrobial resistance phenotype.

With this study, we have now tested the *ahrC* and *eep* loci in animal models of biofilm infection in three mammalian species: rabbits, mice, and now, rats [19, 20]. Our data indicate that *E*. *faecalis* strains with mutations in the *ahrC* and *eep* genes did not significantly differ from the wild-type strain in the rat foreign body osteomyelitis model when inoculated at multiple concentrations. Further, we established that biofilm antimicrobial resistance in *E*. *faecalis* is not dependent on either *ahrC* or *eep*. The role of the transcription factor encoded by the *ahrC* locus in enterococcal virulence and biofilm formation is currently under investigation. Specifically, we anticipate that elucidation of the AhrC regulon will reveal regulatory targets that function in the process of surface attachment. It is important to note the possibility that phenotypic changes unrelated to biofilm formation caused by disruption of either *ahrC* or *eep* may also contribute to virulence in the various animal models of biofilm-associated infection. Therefore, further experimentation is warranted to delineate mechanisms of enterococcal biofilm formation *in vivo*. Additional characterization of these enterococcal biofilm-associated virulence factors may facilitate the identification and development of novel antimicrobial agents or vaccines. Finally, orthologues of *ahrC* and *eep* are present in staphylococci, suggesting that the results of this study may have translational relevance to other leading pathogens of PJI.
